# Riboflavin based setup as an alternative method for a preliminary screening of face mask filtration efficiency

**DOI:** 10.1038/s41598-024-59485-7

**Published:** 2024-04-17

**Authors:** Aida Cavallo, Tamer Al Kayal, Giorgio Soldani, Paola Losi, Lorena Tedeschi

**Affiliations:** Institute of Clinical Physiology, National Research Council, Pisa, Italy

**Keywords:** Biomedical engineering, Health occupations, Assay systems, Biological fluorescence

## Abstract

Face masks are essential in reducing the transmission of respiratory infections and bacterial filtration efficiency, a key parameter of mask performances, requires the use of *Staphylococcus aureus* and specialised staff. This study aims to develop a novel method for a preliminary screening of masks or materials filtration efficiency by a green, easy and rapid setup based on the use of a riboflavin solution, a safe autofluorescent biomolecule. The proposed setup is composed of a commercial aerosol generator commonly used for aerosol therapy, custom 3D printed aerosol chamber and sample holder, a filter for downstream riboflavin detection and a vacuum pump. The filtration efficiency of four different masks was assessed using the riboflavin-based setup and the bacterial filtration efficiency (BFE). The averaged filtration efficiency values, measured with both methods, were similar but were higher for the riboflavin-based setup (about 2% for all tested samples) than bacterial filtration efficiency. Considering the good correlation, the riboflavin-based setup can be considered validated as an alternative method to bacterial filtration efficiency for masks and related materials fabrics filtration efficiency screening but not to replace regulation approaches. The proposed setup can be easily implemented at low price, is more rapid and eco-friendly and can be performed in chemical-physical laboratories without the needing of biosafety laboratory and specialised operators.

## Introduction

Face masks can be defined as protective equipment with the primary function of reducing the transmission of airborne particles or droplets^1^. The use of face masks to prevent the transmission of respiratory infectious agents was limited to healthcare worker or to individuals to reduce the impact of air pollution, allergies, and risk of infection in immunosuppressed people^2^. More recently, with the COVID-19 pandemic, face masks have become widely used to limit the spread Sars-CoV-2 virus because airborne transmission was demonstrated as the main person-to-person transmission way in poorly ventilated indoor environments^3,4^.

Two main categories of face masks are available on the market, surgical or medical masks and face filtering pieces (FFP) regulated by EN 14683:2019 and by standard EN 149, respectively. From the beginning of 2020, there was an increment of face mask requests and therefore new products, known as homemade or community masks^5^, appearing on the market leading to a high demand of asks conformity testing. Instead of surgical and FFP masks, homemade masks are not regulated and are made with a large variety of textiles as well as with diverse designs in terms of shape and number of overlapped textiles layers^6,7^.

Despite the demonstrated relation among mask design features and fitting with air leakage and overall filtration performance^8,9^, the EN 14,683:2019 for surgical masks indicate the Bacterial Filtration Efficiency (BFE) as a key parameter of filtration efficiency of the face mask^10^. The BFE assessment is based on the use of *Staphylococcus aureus* aerosol and requires specific instrumentation such as Biosafety Level 2 laboratories, a careful evaluation of the biological hazard risk as well as specialized staff in handling of *S. aureus.* The use of pathogenic bacterium limits the access to the methodology therefore alternative methods for filtration efficiency assessment have been described in literature. Chiera et al. proposed the use of a less pathogenic bacterium, the *Staphylococcus epidermidis* (Biohazard group 1 microorganism) instead of *S. aureus* for the BFE assessment demonstrating the results validity and turnaround time^11^. Whyte et al. showed a correlation between BFE and particle filtration efficiency (PFE) required for FFP mask. In particular, the PFE is a relatively easier alternative due to the non-biological aerosol employed^1^.

The present study aims to implement a setup for a preliminary investigation of mask filtration efficiency based on the use of a riboflavin solution aerosol as an easy-to-handle and eco-friendly solution with respect to *S. aureus*. The riboflavin, also known as vitamin B2, is a water-soluble vitamin that possesses unique biological and physicochemical properties. In particular, riboflavin is a good fluorescent emitter and for this property is employed in various luminescent sensing systems^12,13^. The proposed method is an alternative to BFE for a preliminary sceening but not to replace the regulation approaches. The setup validation was performed by comparing the filtration data of four different face masks obtained using the proposed custom setup and BFE data obtained by accredited laboratory. In particular, we hypothesized that the proposed riboflavin-based setup will be able to distinguish between masks or material masks with high (i.e. > 90%) and low (i.e. < 90%) filtration efficiency. Therefore, to assess the potentiality of proposed setup, the study was conducted testing regulated devices with filtration efficiency indicated by UNI EN 14683:2019 and, also, nonregulation or alternative masks.

## Materials and methods

### Face masks sample

**Figure 1:**
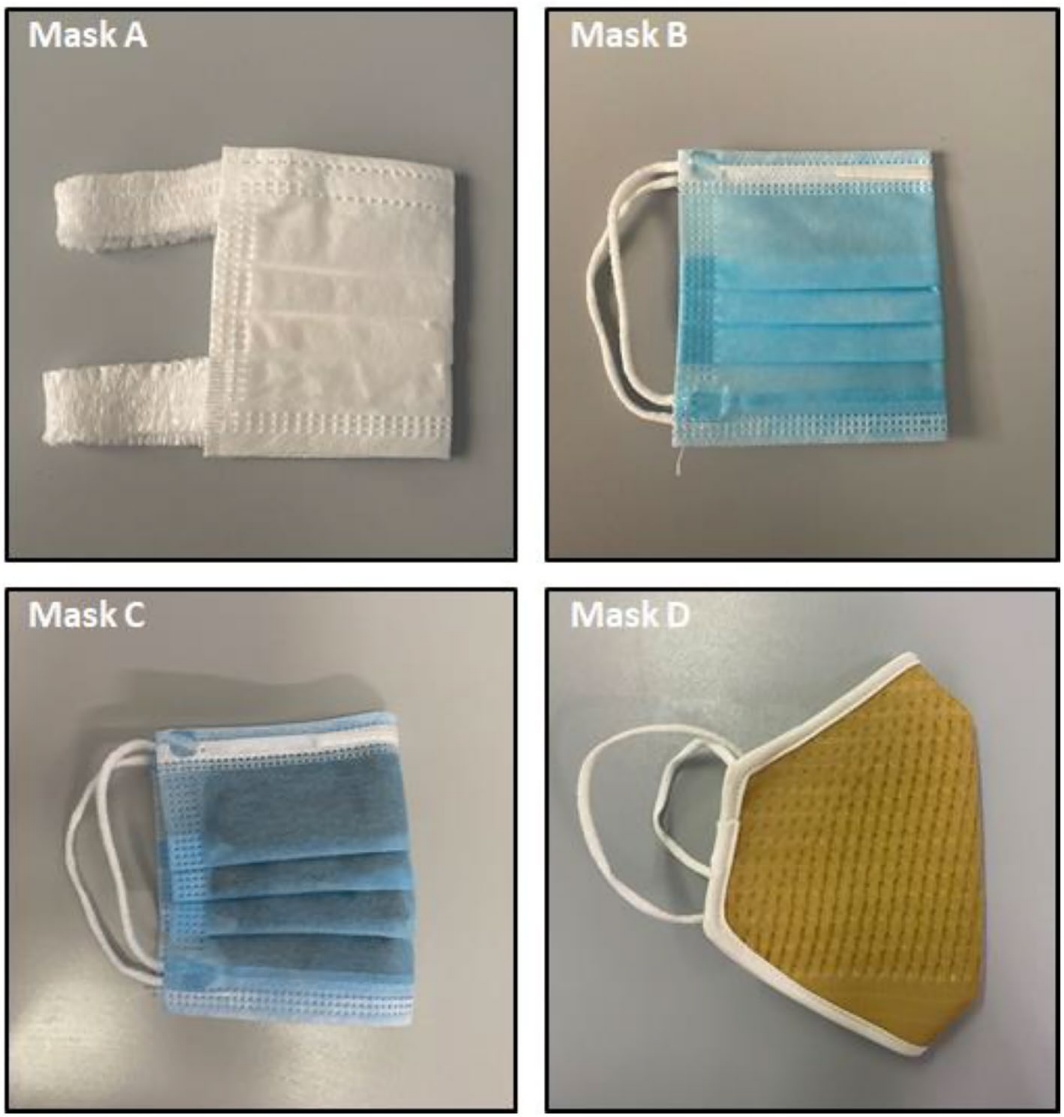
Masks selected for filtration efficacy assessment: (**A**) surgical mask type IIR; (**B**) surgical mask in polypropylene produced in derogation; (**C**) homemade mask in polypropylene with silver antimicrobial coating and (**D**) homemade mask in cotton, copper and polypropylene.

Four different face masks, showed in Fig. 1, were selected for the testing: (i) surgical mask composed by three layers produced in accordance with UNI EN 14683:2019 classified as Type IIR (*Mask A*); (ii) mask composed by three layers of spunbond polypropylene produced during the COVID-19 pandemic in derogation of Italian legislation according to D. lgs. 18/2020 released on 17 March 2020 (*Mask B*); (iii) homemade three-layer mask with the inner and outer layers composed by spunbond polypropylene while the middle layer is spundbond polypropylene with silver based antimicrobial coating as proposed by Ferrari et al.^14^ (*Mask C*); (iv) homemade two-layer mask produced by a local manufacturer and composed by cotton and copper woven fabric outer layer with antimicrobial properties and polypropylene nonwoven fabric 45 g/m^2^ (*Mask D*).

Each sample was conditioned at 21 ± 5 °C and 85 ± 5% of humidity for 4 h before filtration efficiency assessment according to UNI EN 14683:2019.

### Mask morphology

The morphology of the selected masks was evaluated by stereomicroscope (Axio Zoom V16, Carl Zeiss, Oberkochen, Germany) equipped with a z-stack system and digital camera AxioCam 105 (Carl Zeiss) and by scanning electron microscopy (SEM, FlexSEM 1000, Hitachi, Tokyo, Japan). Fiber diameter and layer porosity were estimated using ImageJ 1.52 software (National Institutes of Health, Bethesda, MD, USA).

### Bacterial filtration efficiency (BFE) assessment

The BFE tests were performed by the accredited laboratory according to the standard reported in UNI EN 14683:2019. The BFE is evaluated by a specific setup where an aerosol of *Staphylococcus aureus* cultured in Tryptic Soy broth (mean particle size of 3.0 ± 0.3 μm) is generated and passed through the mask material (100 × 100 mm as sample dimensions and a testing area of at least 49 cm^2^), which is stuck between a six-stage viable aerosol cascade impactor and the aerosol chamber, with a flowrate of 28.3 L/min. The test was performed with the inner layer of the mask in contact with aerosol, using a *S. aureus* suspension between 1.7 × 10^3^ and 3.0 × 10^3^ Colony Forming Unit (CFU) that was nebulized for 1 min and at room temperature. The total duration of each test was equal to 2 min, then the Petri dishes containing Tryptic Soy agar were incubated at 37 °C for 24 h. The BFE is calculated according to Eq. (1):

1 $${\text{BFE}}=\frac{C-T}{C} *100 ,$$

where C is the mean of CFU in two positive controls and T is the total of CFU for each tested sample.

### Riboflavin filtration efficiency assessment

**Figure 2:**
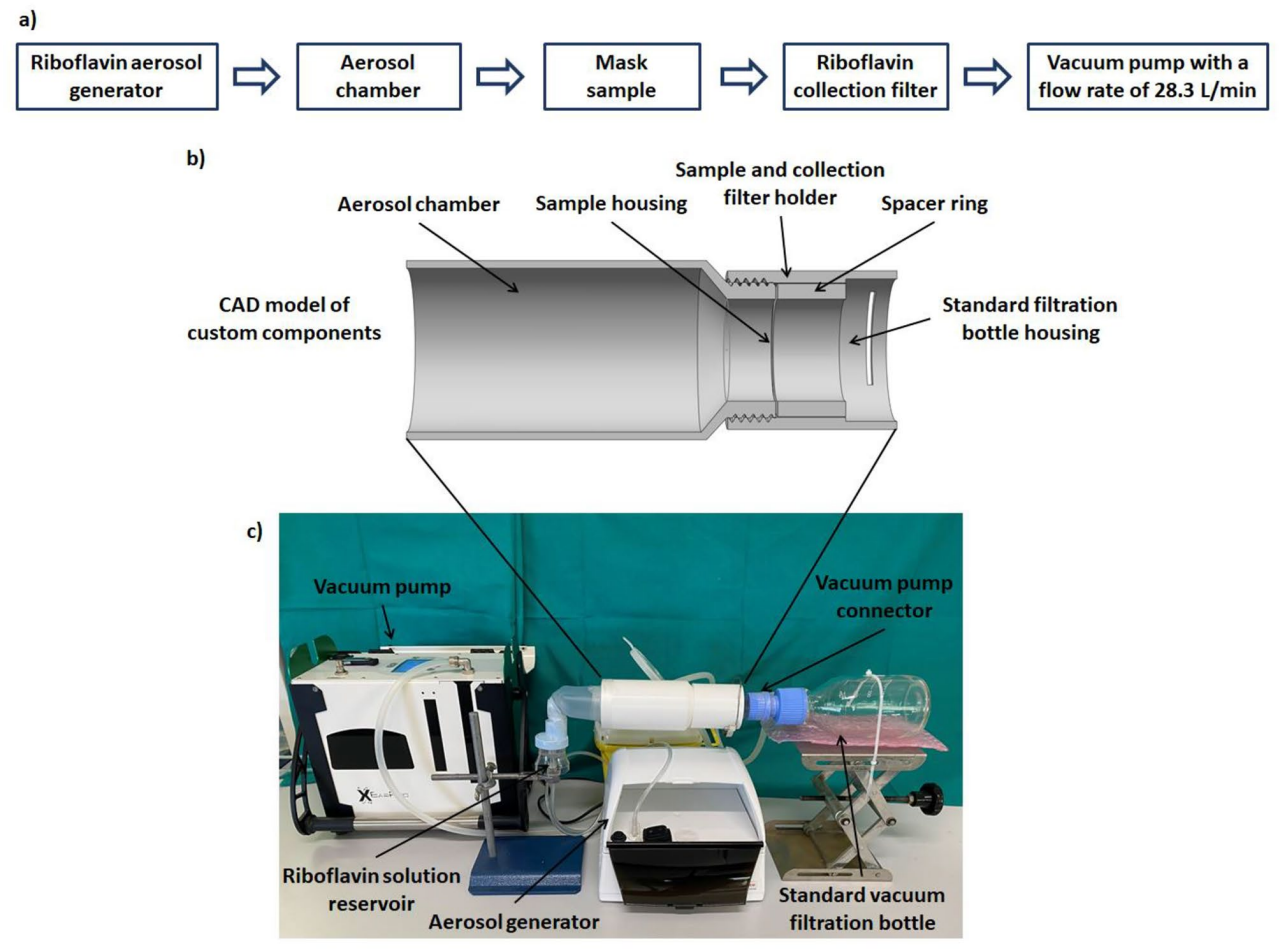
(**a**) Workflow of experimental setup for mask filtration efficiency assessment using riboflavin solution aerosol; (**b**) CAD assembly of setup custom components and (**c**) experimental setup for mask filtration efficiency assessment using an aerosol of riboflavin solution.

The workflow of the experimental setup proposed in this study is reported in Fig. 2a. In particular, the proposed setup is composed of: (i) a commercial aerosol generator (Nebula AirLiquide, Paris, France), commonly used for aerosol therapy in the treatment of respiratory disorders, loaded with 1.25% (w/v) riboflavin solution producing, according to manufacturer instructions,anaerosol size in the range of 0.9–5.1 μm with 50% by volume of the atomized particles less than 1.9 μm; (ii) 3D printed aerosol chamber with internal diameters of 7 cm and length of 11.5 cm (Fig. 2b); (iii) 3D printed sample holder in with a testing area of 12.5 cm^2^ (Fig. 2b) equipped with a spacer ring (thickness of 25 mm); (iv) filter with diameter of 47 mm and pore size of 5 μm (Millipore, Merck KGaA, Darmstadt, Germany) housed on a standard glass filtration assembly for riboflavin aerosol detection downstream of mask sample and (v) vacuum pump (Bulldog, XEarPro, Monza Brianza, Italy) with flowrate of 28.3 L/min and therefore a face velocity of 40 cm/s. The proposed experimental setup is shown in Fig. 2c.

The custom components of setup were designed using Solidworks 3D CAD software (Dassault Systèmes SolidWorksCorp., Waltham, MA, USA) and were fabricated in Polylactic Acid (PLA) by Sharebot Q 3D printer (Sharebot, Lecco, Italy) equipped with filament fusion deposition technology using the following printing parameters: layer thickness of 0.2 mm, printing speed of 3000 mm/min and printhead temperature of 210 °C.

Each test has a total duration of 2 min with 1 min of aerosol generator on and 1 min off. The filtration efficacy (FE) was calculated according to the Eq. (2):

2 $$\mathrm{FE }= 100 -\left(\frac{{R}_{s}}{{R}_{100}}* 100\right),$$

where R_s_ is the riboflavin amount collected on the filter for each tested sample and R_100_ is the mean of riboflavin amount collected on the filter in three independent tests without sample.

Immediately after testing, each filter was submerged in 2 ml of deionized water and after 30 min of continuous agitation in a horizontal shaker (ASAL VDRL 711D, Milan, Italy) at 40 rpm and room temperature, 300 µL of supernatant for each sample were plated in 96 well plate. The fluorescence was measured using a microplate reader (Infinite 200 PRO, Tecan, Männedorf, Switzerland) with gain of 75 and at 440 and 530 nm as excitation and emission wavelength, respectively. The riboflavin amount on each filter was estimated using a linear calibration curve, correlating the riboflavin amount and the fluorescence of riboflavin solution obtained by serial dilutions of riboflavin solution also employed for aerosol. The tests were performed in triplicates for each mask type.

### Statistical analysis

Data are reported as mean ± standard deviation of three independent experiments. The statistical analysis was performed unpaired *t-test* using StatViewTM 5.0 software (SAS Institute, Cary, NC, USA) to compare the data obtained for each tested mask by BFE according to the UNI EN 14683:2019 and by riboflavin experimental setup. p value < 0.05 was considered statistically significant.

## Results

### Face mask morphology

**Figure 3:**
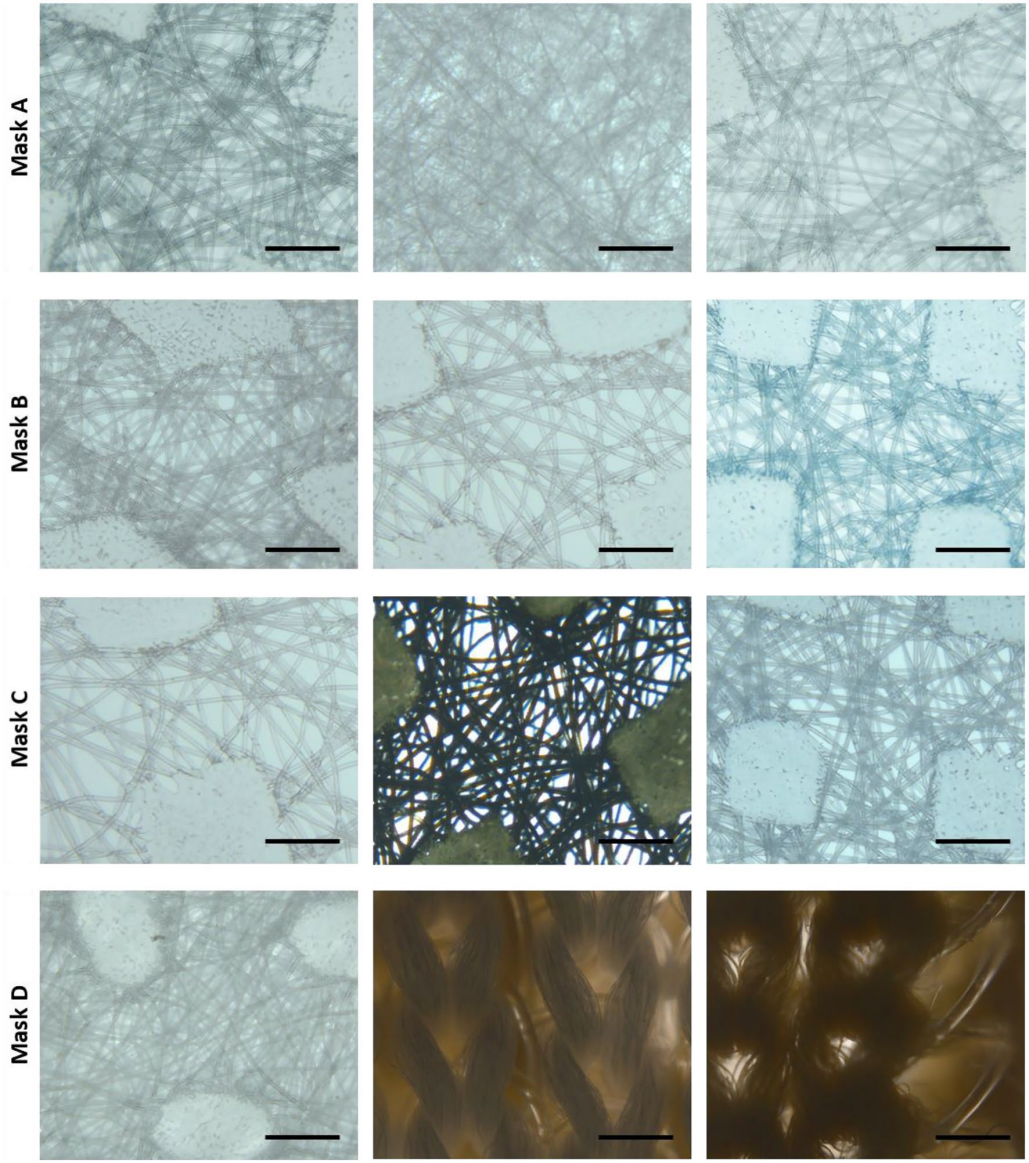
Images captured using a stereomicroscope of mask layers (left images correspond to mouth side); scale bar 500 µm.

**Figure 4:**
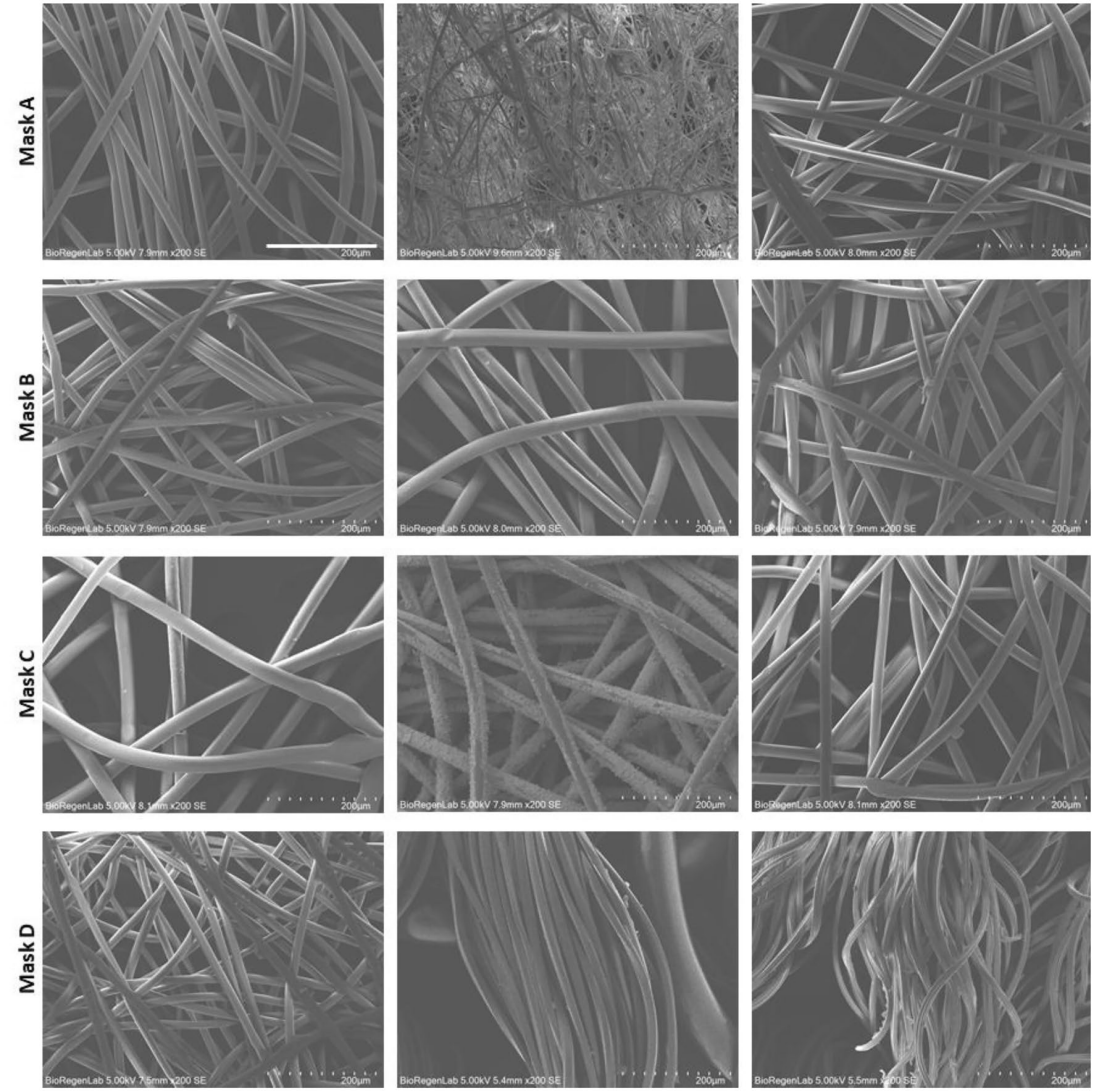
SEM images of mask layers (left images correspond to mouth side); scale bar 200 µm.

Figures 3 and 4 show the morphology of each layer of selected face mask captured using stereomicroscopy and SEM, respectively.

Masks A has spunbound inner and outer layers with porosity of about 16% and fibers diameter of 19.9 ± 1.2 and 18.9 ± 1.2 µm, respectively while the middle layer is a meltblown layer characterised by 3% of porosity and fiber with a diameter of 2.9 ± 1.3 µm. Mask B has three spunbound layers with fiber diameter of 17.6 ± 0.8, 28.1 ± 1.8 and 21.4 ± 2.3 µm, respectively. The middle layer of mask B has an increased porosity, 22%, respect to the inner and outer layers, 18%. The fiber diameter of Mask C inner layer is 29 ± 1.5 µm as well as for middle and outer layers. Mask D is composed of two layers, the inner of non-woven fibers with porosity of 10% and a diameter of 17.4 ± 2.6 µm while the outer one is a woven textile composed of woven copper textile and woven cotton layers held together by a polyester wire.

### Bacterial filtration efficiency (BFE) tests

**Figure 5:**
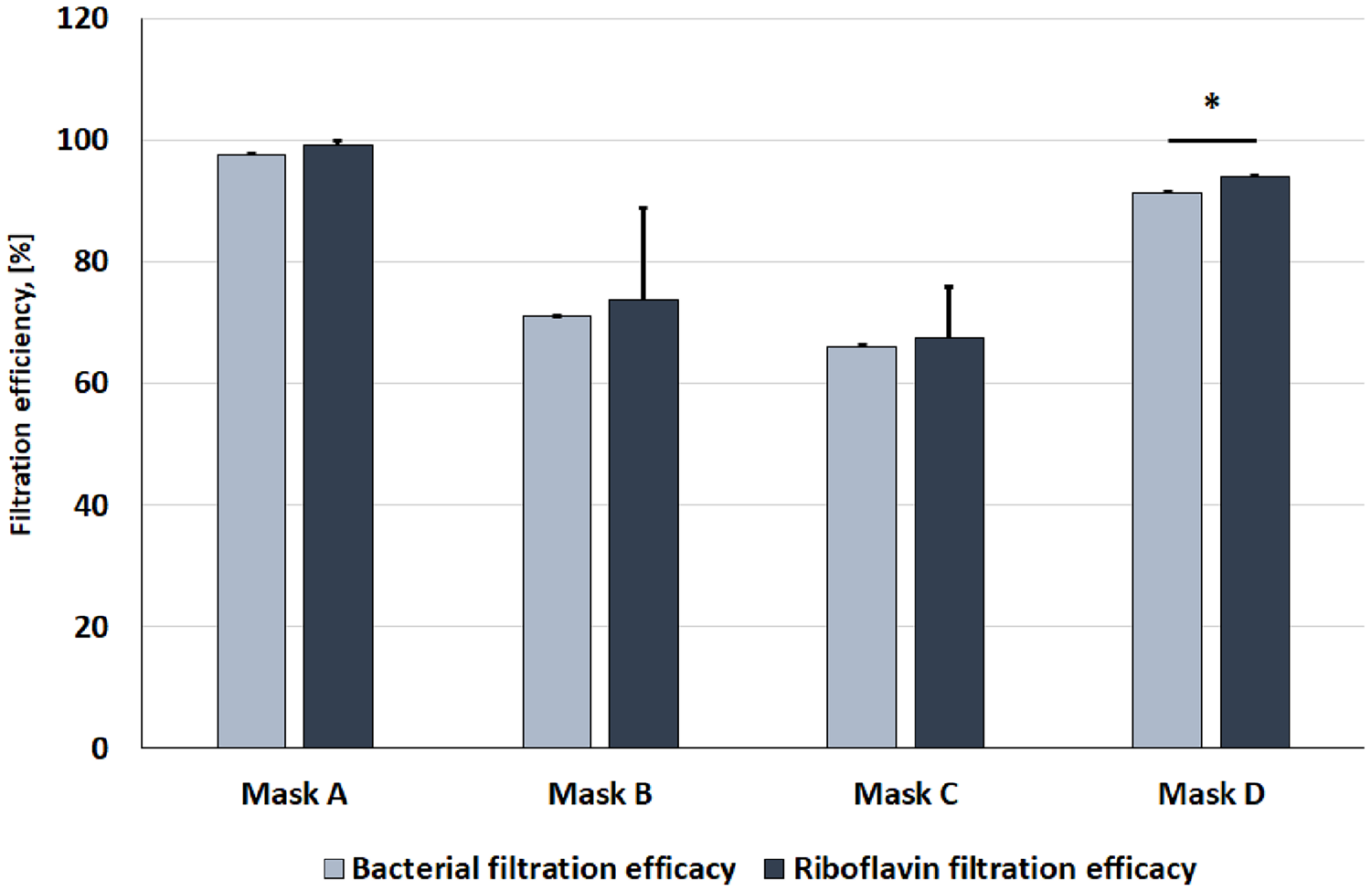
Comparison of data on filtration efficiency obtained by bacterial filtration efficiency (BFE) and by riboflavin experimental setup. Data presented as mean + standard deviation of three independent experiments. *p value < 0.05.

The BFE values measured for tested masks are reported in Fig. 5.

### Riboflavin filtration efficiency assessment

**Figure 6:**
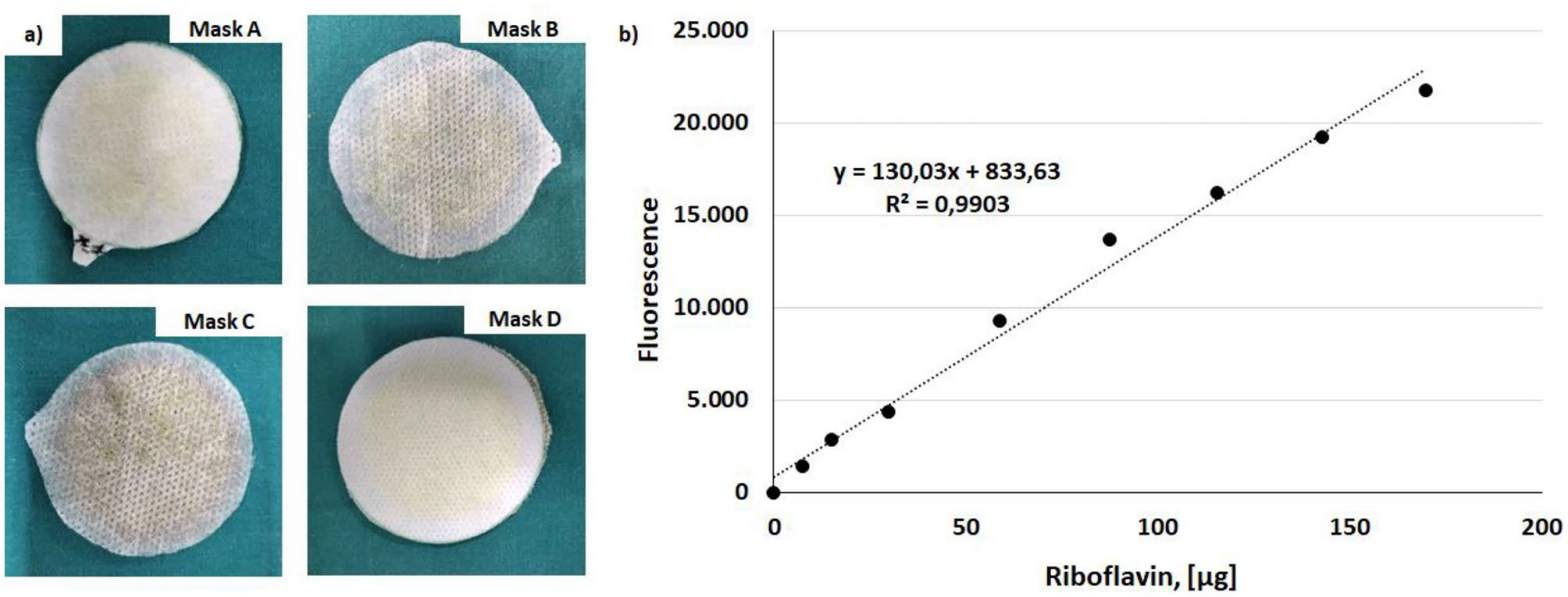
(**a**) Representative images of masks samples immediately after testing; (**b**) linear calibration curve to estimate the detected riboflavin amount downstream of mask sample; fluorescence measured with a gain of 75 and at 440 and 530 nm as excitation and emission wavelength, respectively.

Representative images of the tested masks samples immediately after testing are reported in Fig. 6a. The linear calibration curve correlating riboflavin amount and solution fluorescence employed to analyse fluorescence data related to the tested samples is reported in Fig. 6b. The calculated values of riboflavin filtration efficiency are reported in Fig. 5.

### Data comparison

Comparison of data on filtration efficiency (Fig. 5) obtained by BFE and by riboflavin experimental setup was performed to validate the method proposed in this study. Except for the filtration efficacy of mask D, no statistically significant differences were observed between the data obtained using both described methods for filtration efficacy assessment.

## Discussion

With the Sars-CoV-2 virus diffusion, considering the significative reduction of virus transmission associated with the mask wearing, great attention was focused on new masks development as well as on the mask filtration performance assessment^15–18^. In this context, the aim of proposed study is to develop a new tool for a green, easy and rapid preliminary screening of mask and related materials fabrics filtration efficiency. In particular, the present method should be used to distinguish between masks or materials (i.e. fabrics, tissue non-tissue or their combinations) with high (i.e. > 90%) and low (i.e. < 90%) filtration efficiency, therefore, not to replace the regulation approaches.

The proposed setup is composed of a commercial aerosol generator commonly used for aerosol therapy, custom components, such as the aerosol chamber and the sample holder fabricated using 3D printing as a labware prototyping technology, collection filters, a standard glass filtration assembly and a vacuum pump with a flow rate of 28.3 L/min. In addition, the airborne transmission is simulated using a riboflavin aqueous solution which is an autofluorescent, eco-friendly and easily-to-handle biomolecule solution. The pore size, 5 µm, of the collection filter employed for riboflavin detection downstream of the mask sample is a key aspect of the proposed setup because pores of lower diameter did not allow to maintain the right pump flow rate (i.e. 28.3 L/min) during the test and higher pore size could cause loss of riboflavin solution and therefore of detected information.

Moreover, preliminary experiments (data not shown) were conducted in order to assess the extraction efficiencies of riboflavin from the collection filter in 30 min of continuous agitation demonstrating that the filter is able to release the total collected riboflavin amount when submerged in water. As indicated in UNI EN 14683:2019, each test was carried out by generating the aerosol for 1 min while the vacuum pump was active for two minutes with the recommended flow rate of 28.3 L/min. The sample size (12.5 cm^2^), which is strictly correlated to the available glass filtration assembly (diameter 47 mm), combined with 28.3 L/min of flow rate generates a face velocity of 40 cm/s with respect to a maximum of 10 cm/s indicated by regulation. Schilling et al. (2021) investigated the role of face velocity in filtration efficiency demonstrating that increased face velocities (up to 25 cm/s) resulted in a reduced filtration efficiency, but faster flow rates may provide more potential to discern between masks^19^. Moreover, in FDA-BFE, only the flow rate of 28.3 L/min without sample dimensions specification is reported^20^.

Surgical masks can be classified as type I for BFE values ≥ 95% and type II or IIR for BFE values ≥ 98%. Among the tested masks of this study, only mask A is compliant with the UNI EN 14683:2019. The same result was obtained for the filtration efficiency assessed using the proposed riboflavin-based setup. Aerosol particles are blocked by masks according to different mechanisms, such as straining, inertial impaction, interception, diffusion, and electrostatic attraction^21^. However, filtration efficiency is strongly correlated with mask fiber diameter and porosity. The high filtration efficiency of mask A (i.e. 97.5% as BFE or 99.2% as Riboflavin filtration efficiency) is related to the meltbown layer which is composed of micro- and nano-fibres with fibre density clearly higher than the other two exterior layers^22^. Mask D inner layer is composed by microfiber of lower diameter respect to the same layer of mask A but the fiber diameter combined with the higher thickness (about 400 µm) of the layer which corresponds to layer thickness used in commercially available FFP2 mask^23^, allowed obtaining good filtration efficiency also for the mask D (i.e. 91.4% as BFE or 93.9% as Riboflavin filtration efficiency).

Despite BFE test represent the standard for filtration performance assessment, several scientific questions remain unanswered and possible modernization of the test methods should be considered such as the mask fits on the face to prevents leakage^24,25^.

In literature, there are studies focused on alternative methods to BFE for filtration efficiency assessment especially on the use of inert particles instead of biological ones^17,26–29^ but only few of them considered the comparison with BFE^1,11,17^. In this study, the comparison between the filtration efficiency data obtained by both methods on the same samples was performed with the aim to determine if BFE could be predicted from riboflavin filtration efficiency. If only the average filtration efficiency is considered, the proposed method allowed to obtain data quite similar to the BFE. Riboflavin filtration efficiency is higher than the BFE for all tested samples with an average difference of 2.1%. Despite this, no statistically significant differences were observed for the mask A, B and C while the data obtained for the mask D are statistically significant (p < 0.05) confirming that riboflavin filtration efficiency can predict BFE but not replace it.

He et al. in their study reported that the filtration efficiency of surgical mask is affected by aerosol particle dimensions^17^. In the present study, riboflavin aerosol particle size is more distributed with respect to the *S. aureus* aerosol (0.9–5.1 μm vs 3.0 ± 0.3 μm). However, the combination of riboflavin aerosol with higher face velocity respect to BFE allowed obtaining data quite similar to regulation approaches.

 Moreover, the aerosol chamber of the proposed setup has a lower length respect to the chamber described in UNI EN 14683:2019. The reduced distance between aerosol generator and sample could be responsible of high measured value of filtration efficiency.

The proposed riboflavin-based setup has the main advantage of using the aerosol of a natural, safe eco-friendly, and easy-to-handle solution of riboflavin respect to the bacteria aerosol required by UNI EN 14683:2019. In addition, the riboflavin detection downstream of the mask sample can be performed using a filter and a plate reader respect to the complex equipment required by setup which employs solid or inert aerosol particles^17,26–29^. This approach can be beneficial also during materials manufacturing, before mask assembly, to quickly evaluate if the fibre network and the filtrating power is ensured for every batch, allowing prompt corrective actions when needed.

Considering the good correlation between the averaged values of riboflavin filtration efficiency and BFE, the proposed method can be used as an alternative method to BFE to predict filtration efficiency for masks with high (> 90%) aerosol retention capacity such as masks A and D as well as for masks with lower (< 90%) retention capacity such as masks B and C. In particular, the riboflavin-based setup can be employed for a preliminary screening of masks and related materials. In conclusions, the proposed setup can be easily implemented purchasing on the market the riboflavin tabs at low price as well as the other components of the setup are easily accessible. Moreover, the riboflavin filtration efficiency assessment is more rapid than BFE, 30 min vs. 24 h, and can be performed in general lab without the needing of biosafety laboratories and specialised operators.

More investigations should be performed on samples which have values of riboflavin filtration efficiency similar to the cut off of 95% and 98% indicated by UNI EN 14683:2019 for type I and type II surgical mask, respectively. As BFE assessment, we evaluated the filtration efficiency on mask material without considering the face mask fitting which is crucial for real protection. An improvement of the proposed setup could consider face mask wearing simulation like in standard EN 149.

## Data Availability

The datasets used and/or analysed during the current study are available from the corresponding author on reasonable request.
